# Supplementary material for: Glycomacropeptide Safety and Its Effect on Gut Microbiota in Patients with Phenylketonuria: A Pilot Study
Source: Nutrients. 2022 Apr 29;14(9):1883. doi: 10.3390/nu14091883 (PMC9104775; doi:10.3390/nu14091883)
Supplement: Supplementary file 1 [file nutrients-14-01883-s001.zip › nutrients-1682872-supplementary.pdf]

Supplementary Table S1. Median values and interquartile ranges (IQR) of nutritional values. Statistical significance was determined through a non-parametric Wilcoxon paired signed-rank test; p-values below 0.05 were considered as significant.

Nutritional Variable	Median value at T0; IQR (n=9)	Median value at T1; IQR (n=9)	*p*-value
**Energy**			
Kcals/day	1690; 101	1825; 628	0.820
**Protein**			
% en	15.6; 2.7	13.8; 4.1	0.301
g/day	73.5; 16.4	68.3; 17.1	1
g/day (from PS)	53; 10.4	56.7; 12.7	0.652
%GMP	0; 0	79.3; 47.9	0.009
% AA	100; 0	20.7; 47.9	0.009
Phenylalanine (mg/day)	608.6; 588.8	905; 328.3	0.359
**Carbohydrate**			
% en	57.6; 4.1	57.2; 4.5	0.570
**Lipids**			
% en	27.5; 5	26.8; 4.3	0.250
**Fiber**			
Overall (g/day)	23.9; 8.4	23.9; 5.7	1
soluble fiber (g/day)	1.3; 0.9	1.9; 0.4	0.250
**Vitamins and minerals**			
Vit B12 (µg/day)	10.7; 2.6	5.4; 7;0	0.008
Folate (µg/day)	487.4; 101.0	339.4; 267.6	0.164
Vitamin D (µg/day)	28.0; 12.5	22.5; 5.2	0.203
Calcium (mg/day)	2038.4; 524.7	1392; 535.7	0.039
Phosphorus (mg/day)	1189.7; 318.2	1310.0; 327.9	0.203
Iron (mg/day)	19.6; 9.6	18.7; 6.8	0.910
**Glycemic index**	64; 6.9	68.8; 5.5	0.074
**Glycemic load**	177; 49.1	222.8; 104	0.820

Supplementary Table S2. Fecal SCFAs concentrations (µmol/mg)

	**Median value at T0 (IQR)**	**Median value at T1 (IQR)**	***p*-value**
Acetate	25.8 (13.9)	31.6 (34)	0.432
Propionate	20.1 (9.2)	20.3 (14.6)	0.432
Butyrate	12.7 (12.4)	9.8 (9.9)	0.492
Isobutyrate	1.5 (1.2)	1.8 (1.3)	0.922
Isovalerate	1.5 (0.7)	1.9 (2.4)	0.477

Supplementary Figure S1. SCFA quantification among patients. Paired boxplot of the SCFA analyzed, reporting the trend for each patient through the time of GMP intervention. No significant differences were observed.

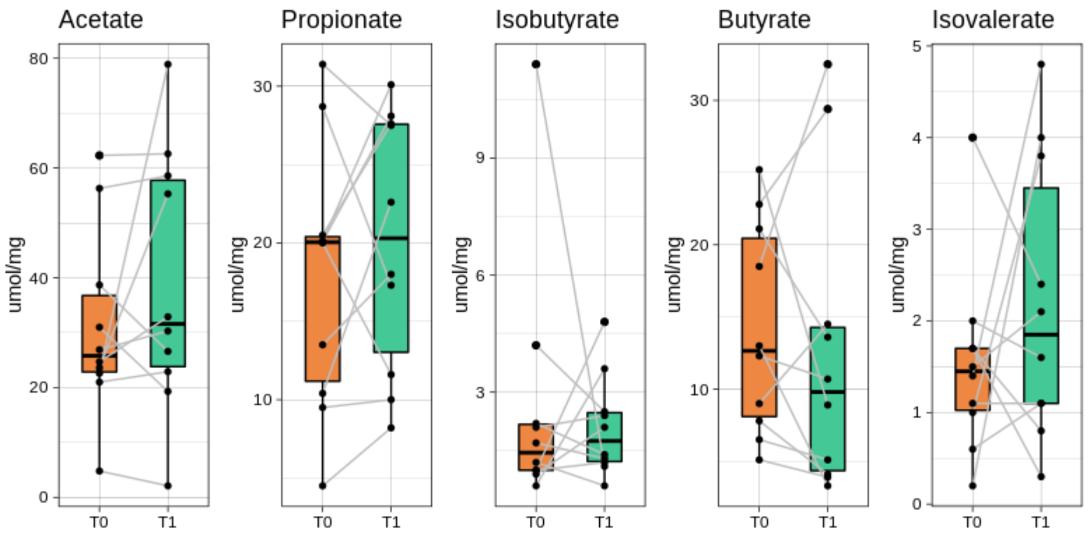

Supplementary Table S3. Taxonomy relative abundances at T0 and T1. Bacterial relative abundances are reported at phylum, family, and genus phylogenetic levels (expressed as median and interquartile range). Statistical differences were evaluated through a non-parametric Wilcoxon paired signed-rank test; only significant p-values ($p<0.05$) are shown.

**Taxonomy**			**Median (IQR)**		***p-value***
**Phylum**	**Family**	**Genus**	**T0**	**T2**	
Firmicutes			56.75 (18.53)	75.86 (28.04)	
	Ruminococcaceae		22.88 (15.9)	40.22 (35.6)	
		Subdoligranulum	7.04 (11.99)	17.66 (15.88)	
		Faecalibacterium	3.56 (5.38)	4.95 (8.84)	
		Ruminococcus 2	1.01 (4.13)	0.84 (1.81)	
		[Eubacterium] coprostanoligenes gr.	1.42 (1.94)	0.02 (0.17)	
	Lachnospiraceae		23.61 (11.11)	26.41 (9.78)	
		Blautia	6.39 (7.91)	4.07 (4.08)	
		Lachnospiraceae NK4A136 group	1.57 (3.65)	2.32 (3.27)	
		Lachnoclostridium	2.12 (0.5)	1.33 (0.4)	
		Anaerostipes	0.73 (1.35)	0.65 (1.4)	
		uncultured (Lachnospiraceae)	1.08 (1.15)	0.49 (0.29)	0.023
		Eisenbergiella	0.72 (1.21)	0.16 (0.87)	
		Roseburia	0.33 (0.46)	0.23 (0.67)	
		Agathobacter	0.23 (0.45)	2.61 (3.41)	0.008
	Acidaminococcaceae		1.78 (4.81)	0.44 (2.6)	
		Phascolarctobacterium	0.33 (3.18)	0.12 (1.11)	
	Veillonellaceae		1.18 (1.71)	0.61 (1.39)	
		Veillonella	0.02 (0.19)	0.09 (0.25)	
		Dialister	0.03 (1.44)	0.52 (1.18)	
	Streptococcaceae		0.34 (0.54)	0.26 (0.75)	
		Streptococcus	0.34 (0.54)	0.26 (0.75)	
Bacteroidetes			29.56 (19.73)	15.49 (20.73)	
	Bacteroidaceae		21.75 (20.02)	13.64 (19.38)	
		Bacteroides	21.75 (20.02)	13.64 (19.38)	
	Tannerellaceae		0.97 (1.55)	0.4 (1.87)	
		Parabacteroides	0.97 (1.55)	0.4 (1.87)	
	Rikenellaceae		1.6 (1.77)	0.75 (1.51)	
		Alistipes	1.6 (1.77)	0.75 (1.51)	
	Prevotellaceae		0.00 (0.03)	0.00 (0.18)	
Verrucomicrobia			2.26 (14.78)	0.01 (0.13)	
	Akkermansiaceae		2.26 (14.78)	0.01 (0.13)	
		Akkermansia	2.26 (14.78)	0.01 (0.13)	
Actinobacteria			3.24 (3.37)	6.61 (5.35)	
	Bifidobacteriaceae		2.09 (2.54)	2.09 (6.25)	
		Bifidobacterium	2.08 (2.53)	2.08 (6.25)	
Proteobacteria			0.97 (1.57)	1.38 (2.58)	
	Enterobacteriaceae		0.24 (0.52)	0.7 (1.33)	
		Escherichia-Shigella	0.24 (0.52)	0.7 (1.33)	
